# Feline Cryptococcosis due to *Cryptococcus gattii* VGII (Recently Renamed as *C. deuterogattii*) in an FIV-Positive Cat With Demodicosis From Manaus, Central Amazon, Brazil

**DOI:** 10.1155/crve/8368783

**Published:** 2025-05-22

**Authors:** Nayara de Fátima Lazameth-Diniz, Adriana Oliveira da Silva Queiroz, Flávia da Silva Fernandes, João Fernando Vieira Ennes, Naira Sulany Oliveira de Sousa, Ana Claudia Alves Cortez, Jacqueline da Silva Batista, Eveleise Samira Martins Canto, Kátia Santana Cruz, Ani Beatriz Jackisch-Matsuura, Ormezinda Celeste Cristo Fernandes, Suanni Lemos de Andrade, Érica Simplício de Souza, Hagen Frickmann, João Vicente Braga de Souza

**Affiliations:** ^1^Laboratório de Micologia, Instituto Nacional de Pesquisas da Amazonia, Manaus, State of Amazonas, Brazil; ^2^Coordenação de Biodiversidade (COBIO), Instituto Nacional de Pesquisas da Amazonia, Manaus, State of Amazonas, Brazil; ^3^Laboratório de Micologia e Bioensaios (LAMIB), Universidade Federal do Oeste do Para, Santarém, State of Pará, Brazil; ^4^Medical Mycology Laboratory, Fundacão de Medicina Tropical Doutor Heitor Vieira Dourado, Manaus, State of Amazonas, Brazil; ^5^Laboratório de Diversidade Microbiana com Importância para Saúde, Instituto Leonidas e Maria Deane Fiocruz Amazonia, Manaus, State of Amazonas, Brazil; ^6^Escola Superior de Ciências da Saúde, Universidade do Estado do Amazonas, Manaus, State of Amazonas, Brazil; ^7^Escola Superior de Tecnologia, Universidade do Estado do Amazonas, Manaus, State of Amazonas, Brazil; ^8^Department of Microbiology and Hospital Hygiene, Bundeswehr Hospital, Hamburg, Germany; ^9^Institute for Medical Microbiology, Virology and Hygiene, University Medicine, Rostock, Germany

**Keywords:** *C. deuterogattii*, cryptococcosis, *Cryptococcus gattii*, feline immunodeficiency virus, fluconazole, molecular genotyping

## Abstract

This case report documents the diagnosis and successful treatment of cryptococcosis caused by *Cryptococcus gattii* VGII in a 20-month-old male domestic shorthair cat from Manaus, Brazil, which was concurrently infected with feline immunodeficiency virus (FIV) and diagnosed with demodicosis. The cat presented with mucopurulent nasal discharge, cutaneous lesions on the neck, and a subcutaneous mass between the shoulder blades. Laboratory investigations, including fine-needle aspiration cytology, fungal culture, and PCR-RFLP genotyping, confirmed the presence of *C. gattii* VGII. The cat was treated with fluconazole (10 mg/kg/day) and topical fluralaner–moxidectin, resulting in complete clinical resolution of all lesions and associated symptoms. This report underscores the significance of considering cryptococcosis as a differential diagnosis in immunocompromised cats presenting with cutaneous or respiratory symptoms. Additionally, it highlights the importance of recognizing the Amazon region as an area of environmental prevalence of *C. gattii* VGII, reinforcing the need for awareness regarding its impact on animal health.

## 1. Introduction

Cryptococcosis is a severe systemic fungal infection primarily caused by the species complexes *Cryptococcus neoformans* and *Cryptococcus gattii* [1]. These opportunistic pathogens are commonly found in decaying organic matter and bird droppings, thriving in both urban and rural environments [2, 3]. Cryptococcosis is the most prevalent systemic fungal infection among cats [2, 3]. Infection usually occurs via inhalation of airborne fungal propagules, which can deposit in the respiratory tract, leading to localized or systemic disease [4–6]. In regions like the Amazon basin, environmental factors such as the abundance of decaying wood and tropical climate create favorable conditions for fungal growth, particularly for *C. gattii* [7–9]. Despite its endemicity in this area, documented cases of feline cryptococcosis remain rare, most likely due to underreporting or a lack of diagnostic resources. Notably, molecular studies have traced the origin of the *C. gattii* genotype VGII, responsible for the Vancouver Island outbreak, to South America, including the Amazon rainforest [10].

Feline immunodeficiency virus (FIV) is a well-documented predisposing factor for opportunistic infections in cats, including cryptococcosis. Immunosuppression caused by FIV disrupts the immune system's ability to manage fungal pathogens, facilitating disease onset and progression [4, 6, 11]. Studies have shown that FIV-positive cats are at a significantly higher risk for systemic fungal infections, particularly in regions where *C. gattii* and *C. neoformans* are endemic [11, 12].

This case report details the first documented case of cryptococcosis caused by *C. gattii* VGII (recently renamed *Cryptococcus deuterogattii*) in a domestic cat in Manaus, Amazonas, Brazil.

## 2. Case Description

### 2.1. History and Clinical Presentation

**Figure 1:**
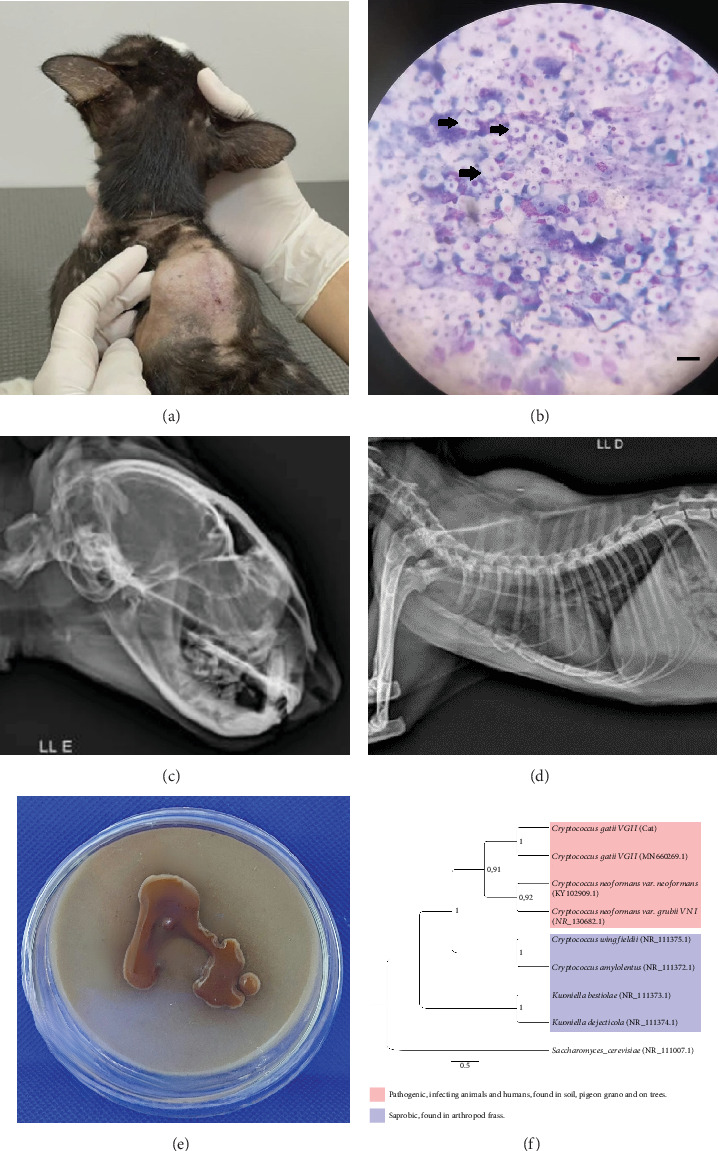
Clinical, radiographic, cytological, mycological, and phylogenetic evidence of cryptococcosis in a domestic cat. (a) Male domestic short-haired cat, aged 20 months, presenting with a subcutaneous nodule between the scapulae. (b) Romanowsky-stained cytology from fine-needle aspiration of the nodule, showing encapsulated yeast-like cells consistent with *Cryptococcus* spp., observed at 400x magnification. This finding is diagnostic of cryptococcal infection. (c) Lateral skull radiograph revealing homogeneous opacity within the nasal cavity, suggestive of an infectious or inflammatory process involving the ethmoid turbinates. This radiographic finding supports a diagnosis of nasal cryptococcosis. (d) Thoracic radiograph demonstrating bronchointerstitial opacification in the caudal lobes, indicative of chronic inflammatory bronchopathy possibly associated with systemic fungal dissemination. The opaque microchip is also visible within the mass. (e) Mycological culture on sunflower seed agar supplemented with chloramphenicol, showing the growth of brown-pigmented colonies after 72 h, consistent with *Cryptococcus* spp. due to melanin production. (f) Phylogenetic tree based on ITS sequencing of the isolate, constructed using maximum likelihood analysis with 1000 bootstrap replicates.

A 20-month-old male domestic shorthair cat, weighing 3.9 kg, was brought to a feline-exclusive veterinary clinic in Manaus, Amazonas, Brazil, in November 2022. The primary complaints were mucopurulent nasal discharge, cutaneous lesions on the neck, and a nodule located dorsally between the shoulder blades (Figure 1). According to the owner, the cat had unrestricted outdoor access.

The cat had been adopted from an animal welfare organization in March 2022. Shortly after adoption, a microchip was implanted (“Animal Microchip RFID Glass Tag,” EM4305, frequency 134.2 kHz, FDX-B, ISO11784/5, size 1.25/7 mm). At the time of adoption, the cat exhibited signs of undernutrition but had otherwise normal physiological parameters.

Approximately 3 months after the microchip implantation, the owner noticed progressive enlargement of a dorsal nodule between the cat's shoulder blades. Concurrently, the cat developed respiratory symptoms. Veterinary assessment confirmed a diagnosis of FIV. The treatment protocol included zidovudine (10–25 mg/kg, orally, twice daily for 90 days, manufactured by Pharmaco [Catswood, Australia], Lot No. 12345) for FIV management and omega-3 supplementation (Ograx, 1000 mg softgel capsules, manufacturer Nutri-Vet [Boise, Idaho, United States]) administered orally once daily for 30 days to improve general health.

Although the FIV treatment led to partial clinical improvement, mucopurulent nasal discharge and cutaneous lesions persisted. Three months after the completion of FIV therapy, the cat still exhibited nasal discharge, a dorsal nodule, and cutaneous lesions, prompting further diagnostic investigation by our veterinary team.

At the time of this assessment, the cat exhibited a soft, subcutaneous swelling located dorsally near the scapulae, measuring approximately 4.0 cm in diameter and 1.5 cm in height. The mass had irregular yet well-defined borders, was firm but nonpainful upon palpation, and remained mobile relative to deeper tissues. There was no skin ulceration or discharge above the nodule, and the overlying skin retained its normal texture and pigmentation. Pulmonary auscultation using a Littmann Classic III Stethoscope (3 M Company, Saint Paul, Minnesota, United States) revealed bilateral crackles. Bilateral mucopurulent nasal discharge was observed, more pronounced in the right nasal cavity, with thick, yellowish discharge leading to mild crust formation at the nostrils. Sneezing episodes occurred approximately two to three times per hour. Cutaneous lesions included alopecic areas on the ventral cervical region (3 × 5 cm) and smaller patches (1–2 cm in diameter) on the hind limbs, displaying mild erythema, scaling, and hyperpigmentation. The skin lesions were nonpruritic and lacked exudate.

Differential diagnoses were considered for each clinical manifestation observed in the cat. For the nasal discharge, potential differentials included upper respiratory tract infections (such as feline herpesvirus or calicivirus), bacterial rhinitis, nasal foreign bodies, and deep fungal infections (*Aspergillus* spp. or *Cryptococcus* spp.). Regarding the subcutaneous nodule, differentials encompassed abscesses, neoplasia (e.g., lymphoma or soft tissue sarcoma), granulomatous infections (including infections caused by *Cryptococcus* spp. and *Mycobacterium* spp.), and sterile inflammatory processes. For the cutaneous lesions, potential causes include ectoparasitic infestations due to fleas or lice, demodicosis, dermatophytosis (due to, e.g., *Microsporum* spp., *Trichophyton* spp.), allergic dermatitis, and other deep fungal infections.

### 2.2. Diagnostic Investigations

Initial laboratory investigations included fine-needle aspiration (FNA) of the nodule using a 22-gauge needle (Becton Dickinson, Franklin Lakes, United States) and a 5 mL syringe (model SecureGrip, Lot No. 7890). The aspirate was stained using Romanowsky stain, revealing a pyogranulomatous inflammatory infiltrate composed of macrophages, neutrophils, and rare yeast-like structures consistent with *Cryptococcus* spp. (Figure 1b). Additional staining with India ink confirmed the presence of a capsule. A potassium hydroxide (KOH, 10%) preparation of skin scrapings, using a standardized 15-min digestion protocol [13, 14] at room temperature, identified *Demodex* spp. mites but no fungal elements as proof of concomitant parasitic infestation.

Serological tests for FeLV antigen and FIV antibodies were performed using a diagnostic assay kit (ELISA; IDEXX SNAP, Brazil Laboratories Ltd, Barueri-SP), confirming FIV positivity and FeLV negativity.

Imaging assessments included skull and thoracic radiographs in ventrodorsal, dorsoventral, and lateral projections (Figure 1c,d).  Figure 1d shows the presence of the microchip inside the mass. Skull radiographs indicated increased opacity in the nasal cavity, consistent with an infectious or inflammatory process, particularly affecting the ethmoid turbinates. Thoracic radiographs showed a diffuse bronchointerstitial pattern in the caudal lung lobes, raising suspicion of chronic inflammatory bronchopathy potentially linked to systemic fungal dissemination.

For mycological identification, the FNA sample was cultured on sunflower seed agar [15] supplemented with chloramphenicol (0.5 g/L, Sigma-Aldrich [Merck, Saint Louis, Missouri, United States], Lot No. 56789). Plates were incubated at 30°C in a humidified incubator (Thermo Scientific, Model IncuFlow-200) with controlled humidity set at 70%, ensuring optimal growth conditions. Colonies were assessed for pigmentation after 72 h (Figure 1e). Micromorphological examination confirmed the presence of encapsulated yeast cells consistent with *Cryptococcus* spp. Genotyping was performed applying polymerase chain reaction-restriction fragment length polymorphism (PCR-RFLP) analysis in line with the protocol by Meyer et al. [16, 17]. In short, DNA was extracted using the phenol–chloroform method [18], and digestion was conducted with *Sau96I* and *HhaI* restriction enzymes [16]. The internal transcribed spacer (ITS) region was sequenced using ITS1 and ITS4 primers in a 50 *μ*L PCR reaction [19]. Amplified products were sequenced on an Applied Biosystems (Waltham, Massachusetts, United States) 3130 Genetic Analyzer as described by the manufacturer.

Phylogenetic analysis, employing the Tamura-Nei model with 1000 bootstrap replicates, confirmed the isolate as *C. gattii* VGII [1]. The ITS sequence was deposited in the NCBI GenBank under accession number MN660269.1. Comparative BLAST analysis demonstrated 98% similarity with *C. gattii* MN660269 (Figure 1e). Minimum inhibitory concentration (MIC) testing was performed following Clinical and Laboratory Standards Institute (CLSI) guidelines M27-A3 [20]. The protocol utilized RPMI-1640 medium (Sigma-Aldrich [Merck, Saint Louis, Missouri, United States], Lot No. 67890) buffered with 0.165 M MOPS (pH 7.0) in sterile 96-well microplates. The plates were incubated at 35°C for 48 h, and MICs were determined using a SpectraMax i3x Microplate Reader (Molecular Devices, San Jose, California, United States). Obtained MIC values for the assessed antifungal drugs were as follows: amphotericin B: 0.062 *μ*g/mL; ketoconazole: 2 *μ*g/mL; itraconazole: 4 *μ*g/mL; and fluconazole: 4 *μ*g/mL, suggesting acceptable susceptibility of the isolate.

### 2.3. Treatment and Follow-Up

The cat was treated with fluconazole (10 mg/kg/day, orally, daily for 90 days) as the primary antifungal therapy [6]. To address the *Demodex* spp. infestation, a single dose of fluralaner and moxidectin (Bravecto Plus Cats [MSD, Rahway, New Jersey, United States], topical application) was administered. Over the treatment period, we followed that the cat showed significant improvement. Nasal discharge was completely resolved, as quantified by visual assessment. Cutaneous lesions and the dorsal nodule resolved completely within 8 weeks of initiating treatment.

## 3. Discussion

Feline cryptococcosis caused by *C. gattii* VGII is rarely reported worldwide, and documented cases from the Amazon region are particularly scarce. Comparisons with similar case reports described in the literature highlight notable differences in clinical presentation and outcomes. For example, recent reports from Southeast Brazil described cases predominantly caused by *C. neoformans*, with clinical signs often limited to respiratory and cutaneous involvement [21]. The uniqueness of the here-presented case lies in the simultaneous occurrence of FIV, cryptococcosis, and demodicosis, along with the availability of genotype information on the causative fungal pathogen.

Several studies support a significant association between FIV infection and the development of cryptococcosis in cats, as immunosuppression caused by FIV facilitates the onset and progression of opportunistic mycoses, including systemic cryptococcal infections. In this case, cats' FIV-positive status likely played a key role in the manifestation and dissemination of cryptococcosis [22, 23]. Moreover, we acknowledge the literature suggesting that FIV infection may also serve as a negative prognostic factor. Some authors have proposed that the proportion of FIV-positive cats among cryptococcosis cases may reflect, or even exceed, their proportion in the general feline population, highlighting a stronger correlation than previously assumed [4, 22–25]. In addition, the presence of demodicosis in this patient reinforces the hypothesis of underlying immunosuppression, since feline demodicosis is uncommon in immunocompetent animals and frequently linked to conditions such as FIV.

The choice of antifungal therapy was guided by the measured MIC values, which suggested likely clinical susceptibility to fluconazole. Although the MIC value (4 *μ*g/mL) was relatively high compared to previous reports [26], fluconazole (10 mg/kg/day) was effective in achieving complete clinical remission. While itraconazole and amphotericin B are also used for the treatment of feline cryptococcosis [6], fluconazole was selected due to its favorable oral administration option and promising reports from the literature [4].

A systemic course of cryptococcal infection was confirmed by both respiratory and subcutaneous manifestations in the here-presented case. Considering the abundance of respiratory symptoms, an initial infection route via inhalation of fungal spores was assumed, which is the most common mode of transmission of *Cryptococcus* species. Secondary cutaneous dissemination is consistent with systemic spread supported by immunosuppression. Whereby, fungal organisms show hematogenous spread from the primary site of infection to skin and subcutaneous tissues [4, 6]. Although available information on environmental exposure was limited, the cat's postadoption outdoor access was most certainly associated with contact to contaminated biomaterials, such as decaying vegetation [4–6].

It is important to highlight that *C. neoformans* and *C. gattii* have distinct environmental niches. While both species are associated with decaying organic matter, *C. neoformans* is notably linked to bird droppings, particularly pigeon excreta, which provide nutrient-rich conditions for fungal proliferation. In contrast, *C. gattii* is more commonly isolated from plant debris, especially in tropical and subtropical regions, including the Amazon. Understanding these ecological differences aids in identifying potential sources of infection and tailoring preventive strategies [7, 10, 27].

The diagnosis of feline cryptococcosis usually relies on a combination of cytological examination and culture [6, 28]. In the case here-presented, PCR-RFLP and the analysis of the fungal ITS were used. Notably, molecular studies have traced the origin of the *C. gattii* genotype VGII—responsible for the severe and persistent outbreak on Vancouver Island—to South America, including the Amazon rainforest [10]. The detection of this genotype in a feline case from Manaus, Brazil, is particularly noteworthy, as it raises concerns about its local circulation and potential implications for public and animal health.

It is also noteworthy that the zoonotic potential of cryptococcosis transmitted by cats is considered extremely low, according to recent studies [25]. Instead, human infections usually result from environmental exposure to fungal propagules [6, 29]. The incubation period for cryptococcosis varies but can range from weeks to several months following initial exposure before clinical signs emerge [6].

Last not least, there is a relatively low number of reported feline cryptococcosis cases in the Amazon region. While limited diagnostic resources may partly explain underreporting, the high likelihood of subclinical colonization in asymptomatic animals could also contribute to this observation. Studies have shown that cats can harbor *Cryptococcus* spp. without showing clinical disease, particularly in regions where the fungus is endemic [6, 28, 29].

This study provides valuable insights into the diagnosis and management of feline cryptococcosis in an endemic region; however, several limitations must be acknowledged. First, respiratory samples were not analyzed, which could have provided additional information on potential pulmonary involvement. Second, we did not perform latex cryptococcal antigen testing (LCAT), which is a recognized diagnostic tool for cryptococcosis; this omission is acknowledged as a study limitation. Third, due to laboratorial limitations, we did not have a detailed discussion on the identification of *Demodex* spp. Additionally, long-term follow-up was not conducted, preventing us from assessing potential late recurrence or chronic disease progression. Future research should focus on expanding diagnostic assessments, incorporating LCAT testing, and conducting longitudinal studies to better characterize the epidemiology of *C. gattii* infections in felines from the Amazon region.

## 4. Conclusion

This study underscores the importance of considering cryptococcosis in the differential diagnosis of immunocompromised cats presenting with cutaneous and respiratory symptoms in endemic regions. Molecular identification and antifungal susceptibility testing were important for accurate pathogen identification and informed therapeutic decisions. Despite limitations such as the lack of long-term follow-up and comprehensive diagnostic evaluations, our findings contribute to the understanding of feline cryptococcosis in the Amazon region.

## Data Availability

The data that support the findings of this study are available from the corresponding author upon a reasonable request.

## General Statement

*Relevance and Novel Information.* This is the first report of cryptococcosis caused by *Cryptococcus gattii* VGII in a cat from Manaus, Amazonas, Brazil. The case was associated with concomitant feline immunodeficiency virus (FIV) infection and diagnosis of demodicosis. Molecular diagnostics allowed pathogen identification on the genotype level, and successful therapy demonstrated the efficacy of fluconazole for feline cryptococcosis. This report contributes valuable data regarding clinical presentation, diagnosis, and management of cryptococcosis in endemic regions, especially in immunocompromised cats.
